# Visible Light-Responsive Platinum-Containing Titania Nanoparticle-Mediated Photocatalysis Induces Nucleotide Insertion, Deletion and Substitution Mutations

**DOI:** 10.3390/nano7010002

**Published:** 2016-12-28

**Authors:** Der-Shan Sun, Yao-Hsuan Tseng, Wen-Shiang Wu, Ming-Show Wong, Hsin-Hou Chang

**Affiliations:** 1 Department of Molecular Biology and Human Genetics, Tzu-Chi University, Hualien 97004, Taiwan; dssun@mail.tcu.edu.tw (D.-S.S.); englishbiology@yahoo.com.tw (W.-S.W.); 2 Nanobiomedical Research Center, Tzu-Chi University, Hualien 97004, Taiwan; 3 Department of Chemical Engineering, National Taiwan University of Science and Technology, Taipei 10607, Taiwan; tyh@mail.ntust.edu.tw; 4 Department of Materials Science and Engineering, National Dong-Hwa University, Hualien 97401, Taiwan; mswong@mail.ndhu.edu.tw

**Keywords:** visible light-responsive photocatalyst, TiO_2_, nanoparticle, DNA mutation, *lacZ* α-complementation

## Abstract

Conventional photocatalysts are primarily stimulated using ultraviolet (UV) light to elicit reactive oxygen species and have wide applications in environmental and energy fields, including self-cleaning surfaces and sterilization. Because UV illumination is hazardous to humans, visible light-responsive photocatalysts (VLRPs) were discovered and are now applied to increase photocatalysis. However, fundamental questions regarding the ability of VLRPs to trigger DNA mutations and the mutation types it elicits remain elusive. Here, through plasmid transformation and β-galactosidase α-complementation analyses, we observed that visible light-responsive platinum-containing titania (TiO_2_) nanoparticle (NP)-mediated photocatalysis considerably reduces the number of *Escherichia coli* transformants. This suggests that such photocatalytic reactions cause DNA damage. DNA sequencing results demonstrated that the DNA damage comprises three mutation types, namely nucleotide insertion, deletion and substitution; this is the first study to report the types of mutations occurring after photocatalysis by TiO_2_-VLRPs. Our results may facilitate the development and appropriate use of new-generation TiO_2_ NPs for biomedical applications.

## 1. Introduction

Antibacterial agents, such as antibiotics and disinfectants, are crucial for personal hygiene, water treatment and food production and in healthcare facilities to control the spread of infectious diseases. The overuse of antibiotics and the emergence of antibiotic-resistant and virulent microbial strains has necessitated the urgent development of alternative sterilization technologies. Despite several advancements in antibiotics research, antibiotic-resistant bacterial infections have become a major clinical challenge worldwide because the frequency of outbreaks and epidemics remains high [1].

Photocatalysts are potentially useful in various settings for reducing pathogen transmission in public environments. Titanium dioxide or titania (TiO_2_) substrates, which are primarily induced using ultraviolet (UV) light, are the most frequently-used photocatalysts for antibacterial applications [2,3,4]. The photon energy excites electrons from the valence band to the conduction band, generating positive holes (electron vacancy) in the valence band. The excited electrons and holes are trapped on the TiO_2_ surfaces. These electrons and holes may then recombine and release energy as light or heat, resulting in inefficient photocatalysis. Alternatively, they may react with atmospheric water and oxygen to yield a reactive oxygen species (ROS), such as hydrogen peroxide, hydroxyl radicals (·OH) or superoxide anions (O_2_^−^) [5]. These ROS are powerful biocides that eliminate pathogenic microorganisms. However, human exposure to UV light at bactericidal levels can considerably damage skin and eye tissues [6,7], which limits the use of conventional UV light-induced TiO_2_ substrates in environments where human exposure may occur. This problem can be resolved by impurity doping TiO_2_ with different elements, such as carbon, sulfur, nitrogen and silver, which shifts the excitation wavelength from the UV region to the visible light region [2,8,9,10,11,12,13,14,15,16,17,18,19]. Simultaneously, this process may also reduce the recombination rates of the electron and hole pairs. Visible light-responsive antibacterial photocatalysts (which have a higher quantum efficiency under sunlight than do UV light-responsive photocatalysts) can be safely used in indoor settings to prevent human exposure to UV light [2,8,9,10,11,13,14,15,16].

**Figure 1:**
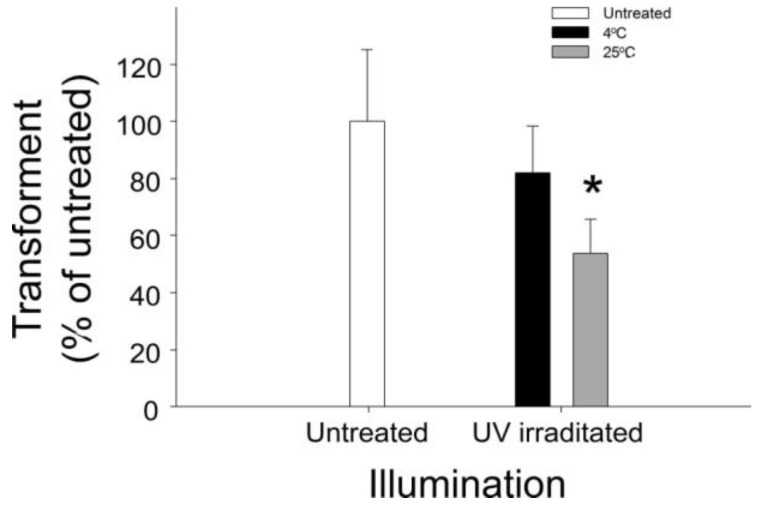
Influence of heat on photocatalyst-induced DNA damage. Plasmid pBlueScript II SK^+^ DNA was transformed to *Escherichia coli* (*E. coli*) competent cells after photocatalysis environments set to 4 °C and 25 °C. The level of DNA damage involving ultraviolet (UV)- and heat-induced nanoscale-TiO_2_ film-mediated photocatalysis was indicated by the reduction of transformants. * *p* < 0.05 vs. 4 °C group. *n* = 6, three experiments with two replicates.

The molecular targets of photocatalysis in bacteria (e.g., DNA, RNA, protein and cell membrane) and the intensity at which these are affected remain unclear. Because photocatalytic reactions involve both oxidation and reduction [20,21], damage observed in the target microorganisms differs from that observed with traditional disinfectants, which involve either oxidation or reduction. This is probably the reason that we previously observed a unique pattern of photocatalysis-induced bacterial destruction [2,10]. UV light-responsive TiO_2_ induces DNA damage without specified temperature control [22,23]; however, UV light alone can also induce DNA mutation and damage [24,25]. In addition, photocatalysis at room temperature (25 °C) induces more DNA damage than that at 4 °C (Figure 1). The absorption of illuminated light energy can produce heat; thus, illumination-induced heat also potentially has a major role in triggering DNA damage. However, visible light-responsive photocatalyst (VLRP)-induced DNA mutations have not been clearly characterized thus far. Therefore, the exact photocatalysis-induced DNA damage, without perturbations of the effects of heat and UV light, warrants further investigation. In the present study, we used a previously-reported visible light-responsive platinum-containing titania (TiO_2_-Pt) photocatalytic nanoparticle (NP) [11,16] to address this question. Our data revealed that VLRPs can induce DNA mutations.

## 2. Results

### 2.1. Involvement of Heat in VLRP-Induced Plasmid DNA Damage

Photocatalysts can absorb light energy and produce heat [26]; simultaneously, heat can also induce ROS production and DNA damage [27]. Herein, we observed that the temperature of photocatalytic films under UV irradiation rapidly increased, easily reaching more than 200 °C. To investigate whether temperature is involved in photocatalytic DNA damage, we used UV-irradiated single-layer TiO_2_ thin films [13] to catalyze plasmid DNA (pBlueScript II SK^+^) in environments at 25 °C and 4 °C. After transforming the photocatalyzed plasmid DNA into competent *Escherichia coli* (*E. coli*) cells, we observed that the experimental samples catalyzed at 4 °C contained considerably more transformants than did those catalyzed at 25 °C (Figure 1). These results suggest that illumination-induced heat also plays a major role in inducing DNA damage.

### 2.2. VLRP Induces Plasmid DNA Damage

**Figure 2:**
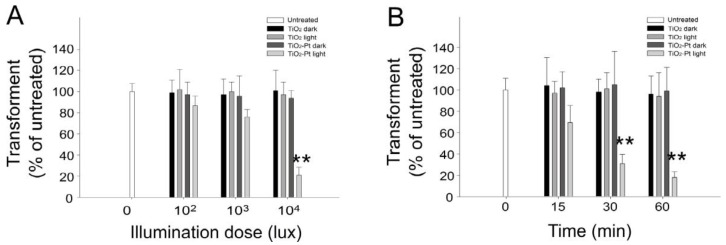
(**A**) Dose-dependent and (**B**) kinetic responses, with increasing illumination density and with increasing time, respectively. The visible light stimulated TiO_2_-Pt photocatalysis-mediated DNA damage was determined by the reduction of transformants. The DNA samples of those dark groups were covered with aluminum foil to prevent the photocatalysis. UV-responsive TiO_2_ NPs were used as control materials. ** *p* < 0.01 vs. respective TiO_2_-Pt dark groups. *n* = 6, three experiments with two replicates.

Both UV light and heat contributed to the DNA damage noted herein; thus, to analyze the DNA damage specifically induced through photocatalysis, we performed a photocatalysis of plasmid DNA using visible light-responsive TiO_2_-Pt NPs as compared to UV-responsive pure-anatase TiO_2_ NPs at 4 °C for 1 h. The results revealed that the number of *E. coli* transformants considerably decreased with the increase in visible light illumination intensity (Figure 2A; TiO_2_-Pt groups), indicating that the DNA damage is induced in a dose-dependent manner. Because light intensity of 10^4^ lux is an effective dose to reduce the *E. coli* transformants (Figure 2A), we used that as the constant illumination intensity with increasing illumination time to obtain a kinetic result. Here, we noted a decrease in the number of transformants, associated with the increasing illumination time (Figure 2B). The DNA samples of those dark groups were covered with aluminum foil to prevent photocatalysis, and thus, no particular response occurred. Because the pure anatase TiO_2_ NPs are UV-responsive, it is reasonable that no DNA damage was observed in the “TiO_2_ light” groups (Figure 2A,B). These results confirm that VLRPs can elicit DNA damage.

### 2.3. Application of VLRP-Induced DNA to Different Plasmids

**Figure 3:**
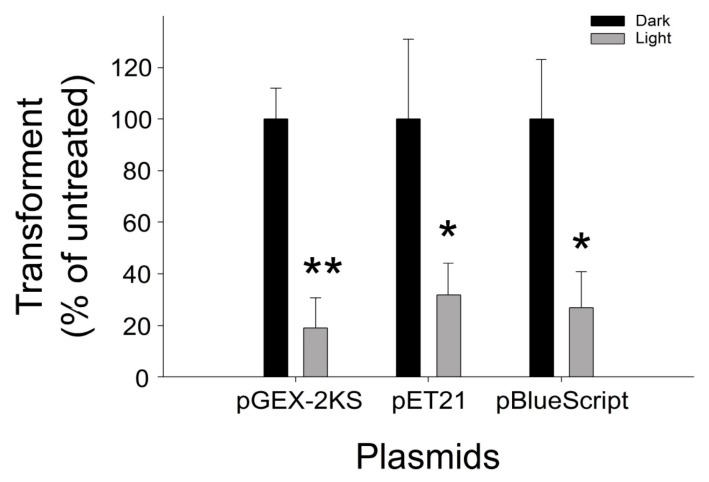
Significant visible light stimulated TiO_2_-Pt-mediated photocatalysis-induced DNA damage occurred in the pGEX-2KS, pET16 and pBlueScript II SK^+^ plasmids. Notably, these plasmids all displayed similar reductions after the photocatalysis. ** *p* < 0.01; * *p* < 0.05 vs. respective dark groups. *n* = 6, three experiments with two replicates.

We subsequently investigated whether our noted visible light-responsive TiO_2_-Pt NP-mediated photocatalysis-induced DNA damage is also applicable to different plasmids. We employed two additional plasmids, pGEM-2KS and pET21, which are used primarily in bacterial recombinant protein expression [28,29,30,31,32,33,34,35,36,37,38], and observed that VLRPs induced considerable damage in all three plasmids, including the pBlueScript II SK^+^ (Figure 3).

### 2.4. VLRP Induces Mutations in Plasmid DNA

**Figure 4:**
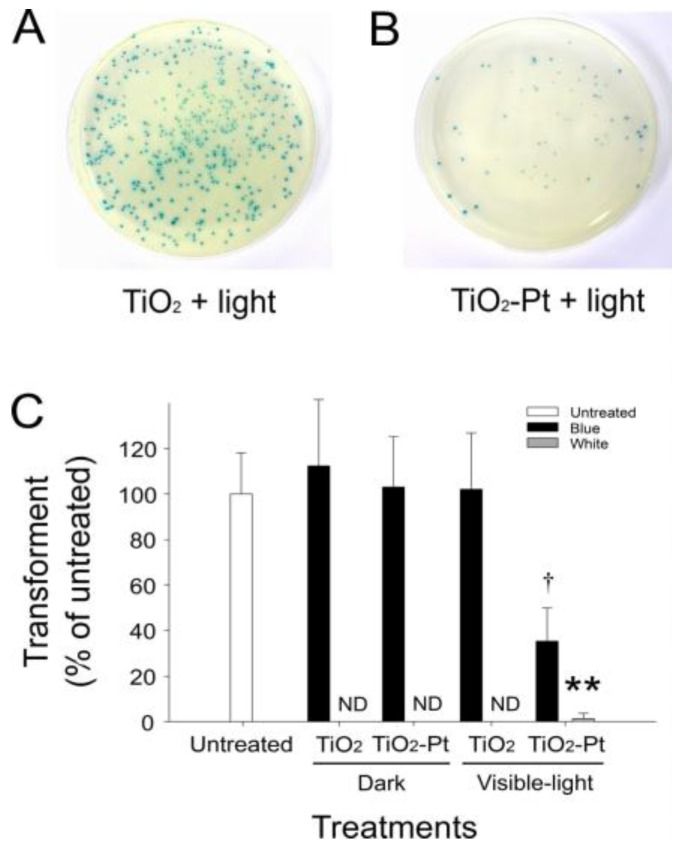
Detection of mutated clones using *lac*Z α-peptide complementation. (**A**,**B**) After being complemented with *lac*Z α-peptide expression, the transformants are displayed as blue colonies on the agar plates with 5-bromo-4-chloro-3-indolyl-β-d-galactopyranoside. The VLRP TiO_2_-Pt NP-mediated photocatalysis markedly reduces the number of transformants, compared with the control groups using UV-responsive TiO_2_ NPs, under visible light illumination. (**C**) Quantified results show that TiO_2_-Pt photocatalysis can induce the formation of white colonies. This indicates that mutations hit the *lac*Zα region because of a loss-of-function (loss-of-complementation) phenotype, compared with the wild-type plasmid-transformed blue colonies. ND: no detected colony. * *p* < 0.05 vs. both blue groups of TiO_2_-visible light and TiO_2_-Pt-dark; ** *p* < 0.01 vs. respective blue groups. *n* = 6, three experiments with two replicates.

To further investigate whether photocatalysis induces mutated DNA damage, we performed an α-complementation analysis of the β-galactosidase gene *lac*Z using pBlueScript II SK^+^ and *E. coli* XL1-blue (Figure S1). Notably, *E. coli* XL1-blue cells with wild-type plasmids expressed functional α-peptide, which complements the defective *lac*Z to produce blue colonies in the presence of the chromogenic substrate 5-bromo-4-chloro-3-indolyl-β-d-galactopyranoside (X-gal) (Figure 4A,B). By contrast, the mutant transformant cells containing damaged DNA in the *lac*Zα region did not produce blue colonies and instead remained white. Thus, using this method, we can differentiate *lac*Zα mutation-type (white) and wild-type (blue) clones. Even at an extremely low frequency (14/2000; 0.7%), visible light-responsive TiO_2_-Pt NP-mediated photocatalysis can induce white colony formation (Figure 4C), indicating that mutations were generated in the *lacZ*α region.

### 2.5. VLRP-Induced DNA Mutations Involve Nucleotide Deletion and Substitution

**Figure 5:**
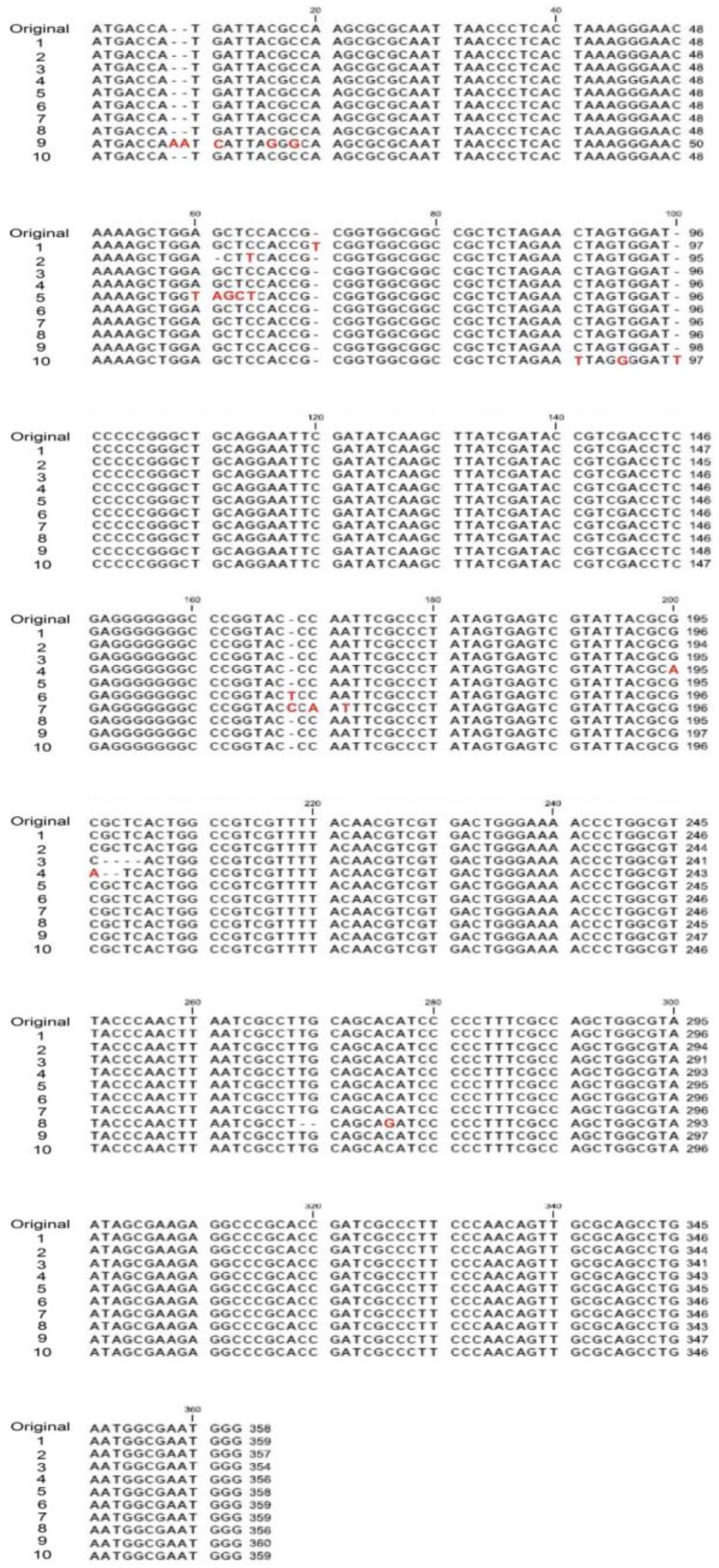
Mutation sites in the *lac*Z α-peptide coding (*lac*Zα) region. Examples of DNA sequences in the *lac*Zα region of the white colonies are provided, in which insertion (Clones 1, 6, 7, 9 and 10) deletion (Clones 2–4, 8) and nucleotide substitution (Clones 2, 4, 5, 7, 8 and 10) mutation types are involved. The nucleotides at the mutation sites are labeled in red.

**Figure 6:**
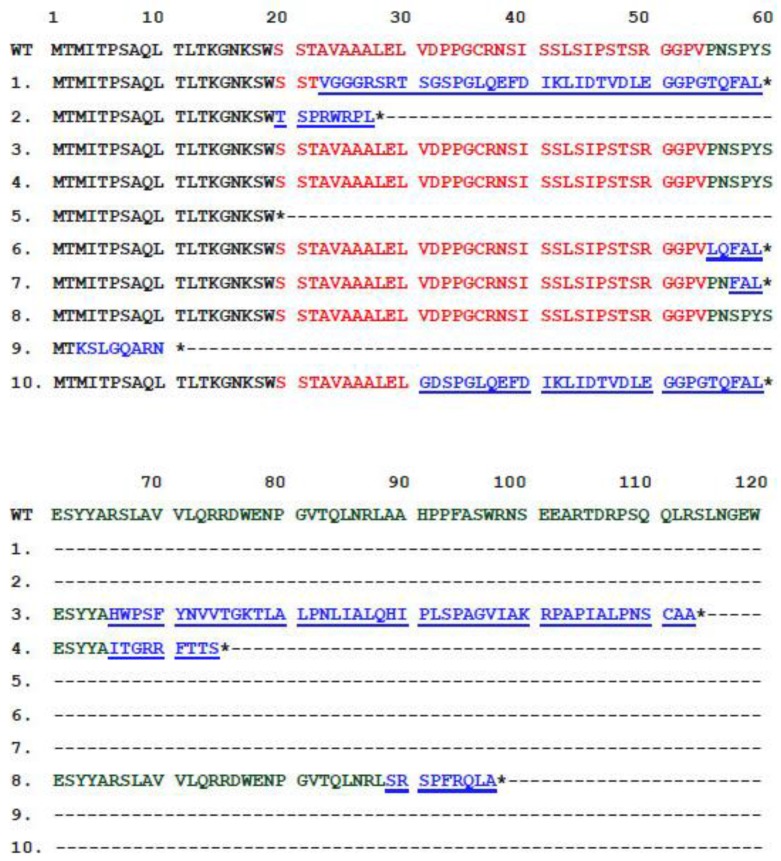
*lac*Z α-peptide amino acid sequence aliment of one wild-type and 10 mutated clones. The amino acid sequences encoded by multiple cloning sites are labeled in red; the sequences encoded by *lac*Zα are labeled in green; and the mutation-caused reading frame shifts are labeled in blue. The * indicates the translation termination by the stop codon.

To further analyze how these mutations were introduced in the plasmid DNA, the *lac*Zα locus on plasmid DNA in the white colony cells was sequenced, and the mutation types and loci were identified. Because of the long sequence deletion, the *lac*Zα region in three clones is likely entirely deleted in 14 isolated white colonies. The remaining 11 colonies contain two clones with identical DNA sequences. Therefore, we analyzed the 10 mutated *lac*Zα DNA sequences. We observed that the mutations included nucleotide deletions and substitutions (Figure 5; Figure S2 with background color), both of which primarily triggered frameshift mutations leading to a loss-of-function phenotype (white colonies) of the α-complementation of *lac*Z, as indicated in the comparison of parental wild-type α-peptide amino acid with the mutant clones (Figure 6). Without exception, all of these mutated plasmids expressed a truncated form of encoded protein (Figure 6).

## 3. Discussion

VLRP-induced DNA mutations have rarely been reported. One study suggested that treatment with TiO_2_ NPs alone (i.e., without light-stimulated photocatalysis) can sufficiently induce mutations in plasmid DNA [39]. By contrast, our data indicate that treatments with both TiO_2_ and TiO_2_-Pt NPs are insufficient to induce DNA damage in darkness (Figure 4), which corroborate several previous reports that TiO_2_ NPs alone cannot damage plasmid DNA in darkness unless photocatalysis is applied [22,23]. Notably, however, the data in these studies were obtained using experimental conditions without temperature control [22,23]. According to our temperature-controlled experiments (Figure 1), such DNA damage can also be attributed to illumination-induced heat. In addition, the aforementioned studies did not examine the mutation types nor locations [22,23]; therefore, the fundamental question regarding the effects of TiO_2_ NP-mediated photocatalysis on the induction of DNA mutations remained elusive.

Our data revealed that treatment with TiO_2_-Pt NP alone is insufficient to induce DNA damage in darkness and that the detectable mutations (i.e., white colonies) can be obtained at an extremely low rate even after VLRP (Figure 4). Some silent mutations, which do not significantly alter the organism phenotype, were also noted outside the *lac*Zα region (data not shown), suggesting that photocatalysis randomly hits different regions of the plasmid. Theoretically, photocatalysis can also introduce mutations and damage into vital regions of the plasmid DNA, such as the replication origin and ampicillin resistance gene loci; therefore, photocatalysis can reduce the transformation rate of a plasmid (Figure 2 and Figure 3).

Here, we observed that the VLRP-induced DNA mutations primarily generated reading frameshifts in all of the mutant clones, except Clone 5 (Figure 5; Figures S1 and S2). Most of these frameshift mutations were caused by photocatalysis-induced insertions and deletions in the plasmid DNA (Figure 5). Because gene expression and protein translation occurs through triplet codons, nucleotide insertion or deletion can shift the reading frame, resulting in the translation of a protein sequence completely different from the original. In addition, frameshift mutations can also introduce an early stop codon (TAA, TGA or TAG). Thus, the translated protein may become abnormally short or long and most likely lose its function. Here, Clone 5 is of particular interest, because the stop codon TAG was directly created at the mutation site, which resulted in the expression of a truncated *lac*Z α-peptide (Figure 6). Consequently, after photocatalysis, a loss-of-function phenotype of these plasmids was noted in the α-complementation analysis.

The chemistry of DNA damage caused by ROS has been well characterized in vitro; for instance, ·OH generates multiple products from all four DNA bases [40,41]. ROS-related DNA oxidation is a major cause of mutations, and it can produce several types of damage, including nonbulky (8-oxoguanine and formamidopyrimidine) and bulky (cyclopurine and etheno adducts) base modifications, abasic sites, nonconventional single-strand breaks, protein-DNA adducts and intrastrand or interstrand DNA crosslinks [42,43,44,45]. In addition, UV light is high-energy electromagnetic radiation that breaks the backbone or cross-link bases (e.g., thymine dimers and pyrimidine dimers: TT or TC) [46,47]. VLRP can also elicit ROS (such as ·OH and O_2_^−^) [5], and even heat can induce the production of ROS, 8-oxoguanine and DNA damage [27]. Therefore, to exclude UV light- and heat-induced DNA damage, we performed photocatalysis using visible light in a temperature-controlled environment. Because the DNA damage was introduced in vitro in our experimental system, the mutation processes of damaged DNA occur in living bacterial cells. DNA damage leads to mutations through three primary pathways: reducing incorporation fidelity, blocking DNA replication and forming frameshifts [48]. UV-responsive TiO_2_-mediated photocatalysis has been demonstrated to trigger DNA double-strand breaks [22,23,49].

Two distinct mechanisms are involved in double-stranded break repair: homologous recombination and nonhomologous end-joining, and both pathways can introduce mutations into DNA [50,51]. Thymine dimers interfere with base pairing during DNA replication, which leads to mutations; thus, translesion DNA synthesis frequently introduces mutations at pyrimidine dimers, both in prokaryotes and in eukaryotes [52]. The C involved in pyrimidine dimers is prone to be deaminated, inducing a C to T transition [53]. As a result, all of the aforementioned mechanisms can potentially cause nucleotide insertions, deletions and substitutions in DNA after exposure to ROS [42,43]; this is likely the reason that we observed such damage in our experiments.

In summary, we investigated the DNA mutations caused by visible light-stimulated photocatalysis at 4 °C, without UV irradiation and heat generation. The results revealed that the photolytic response produced plasmid DNA mutation. As per the loss-of-function phenotype observed in the α-complementation analysis, the mutation types involved nucleotide insertions, deletions and substitutions, which primarily triggered reading frameshifts and the expression of a malfunctioning α-peptide. These results collectively offer novel concepts regarding the safety and potential applications of photocatalytic TiO_2_ materials. For example, because TiO_2_ photocatalysis-mediated DNA damage may cause genotoxicity, caution should be exercised during the synthesis, release and use of photocatalytic TiO_2_ NPs to reduce the environmental impact. By contrast, a DNA damage agent is synergistic with other antibacterial agents, which block different physiological pathways to eliminate pathogenic bacteria [54,55]. Therefore, the genotoxicity of TiO_2_ photocatalysis may facilitate the development of novel antibacterial strategies to manage the spread of pathogens. In short, our research illuminates fundamental knowledge about TiO_2_-NP photocatalysis-mediated DNA mutations and damage, which may be useful for future studies on environmental safety and new-generation TiO_2_-NP development for biomedical applications.

## 4. Materials and Methods

### 4.1. Photocatalyst Preparation

UV light-responsive TiO_2_ thin films were prepared in an ion-assisted electron-beam evaporation system assembled by Branchy Vacuum Technology Co., Ltd. (Taoyuan, Taiwan), as described previously [13]. Visible light-responsive TiO_2_-Pt NPs were prepared through a reduction process using chloroplatinic acid and TiO_2_ NPs as the platinum precursor and pristine photocatalysts, respectively, also as described previously [11]. Next, platinum-containing nanostructured TiO_2_ particles (TiO_2_-Pt) were prepared through a photoreduction process using chloroplatinic acid (H_2_PtCl_6_) and commercial TiO_2_ nanoparticles (ST01; Ishihara, Singapore, Singapore) as the platinum precursor and pristine photocatalysts, respectively. TiO_2_-Pt was prepared by mixing 38 mmol of nonporous TiO_2_ (ST01) and 0.19 mmol of H_2_PtCl_6_·6H_2_O in 100 mL of doubly-distilled water. The TiO_2_ suspension and H_2_PtCl_6_ solution were mixed well using an ultrasonic treatment for 30 min, and the pH value was adjusted to 6 with 0.1 M of NaOH solution using a pH meter (Model 6171, Jenco Instruments, San Diego, CA, USA). Subsequently, a nitrogen stream (100 mL/min) was continuously purged into the reaction chamber to remove oxygen from the solution. The solution was then irradiated with four UV lamps (TUV 10W/G10 T8; Philips Taiwan, Taipei, Taiwan) at an intensity of 1.7 mW/cm^2^ for 4 h. Platinum ions were reduced to platinum metallic nanoparticles by the photo-generated electrons of TiO_2_ and then deposited on the surfaces of TiO_2_. Next, the TiO_2_-Pt particles with a Pt/Ti molar ratio of 0.5% were obtained though centrifugation at 1 × 10^4^ rpm, washed with deionized water and finally dried at 373 K for 3 h. A diffuse-reflectance scanning spectrophotometer (UV-2450; Shimadzu, Kyoto, Japan) was used to obtain the UV-visible absorption spectra of the NPs, which were shown in our previous work [16]. The average particle size and morphology were determined through transmission electron microscopy (Tecnai G2 F20 TEM, FEI, Hillsboro, OR, USA), and the crystal phase of the photocatalyst was identified through X-ray diffractometry with CuKα radiation (λ = 0.154 nm, D/Max RC; Rigaku, Tokyo, Japan). Finally, the material compositions were determined using X-ray photoelectron spectroscopy (SSI-M probe XPS system; Perkin Elmer, Waltham, MA, USA), as described previously [11]. The emission spectra of irradiated UV light and visible light used in this study were analyzed and illustrated (Figure S3).

### 4.2. Bacterial Strains and Culture

*E. coli* XL1-blue (genotype: *recA1 endA1 gyrA96 thi-1 hsdR17 supE44 relA1 lac* [F′ *proAB lacIq Z*∆*M15* Tn*10* (Tet′)]) was maintained and grown in Luria–Bertani (LB) broth or agar (MDBio, Inc. Taipei, Taiwan) at 37 °C using a standard laboratory *E. coli* culture method, as described previously [9,13,56]. The bacteria were stored in 50% glycerol (*v*/*v*) in a culture medium at −80 °C before use. Later, to reactivate the bacteria from the frozen stocks, 25-μL bacterial stock solutions were transferred to a test tube containing 5 mL of freshly prepared culture medium and then incubated at 37 °C under agitation overnight (16–18 h).

### 4.3. Plasmids

Glutathione S-transferase expression plasmid pGEX-2KS [28,30,31,32,33,34,35,36,37,38], His-tag expression plasmid pET16 (Merck, Novagen, Darmstadt, Germany) [29,57,58] and cloning vector pBlueScript II SK^+^ (Takara, Clontech Laboratories, Shiga, Japan) [59] were used in this study. All of the plasmid DNA was isolated using plasmid purification kits, according to the manufacturers’ instructions (Qiagen Taiwan, Taipei, Taiwan). The quantity and quality of DNA were determined by measuring the plasmid absorbance at 260 nm and the absorbance ratio at 260/280 nm, respectively, on a UV-visible spectrophotometer (Hitachi Taiwan, Taipei, Taiwan) [15,60] and Nanodrop spectrophotometer (Thermo Scientific, Wilmington, DE, USA) [61,62]. All relevant standard molecular biological methods were used to amplify, purify and store the plasmids [56].

### 4.4. Photocatalytic Reaction of Plasmid DNA

First, plasmid DNA was dissolved and adjusted on a 2 µg/10 µL of Tris-HCl (pH 7.5) buffer. In the UV light-responsive photocatalysis experiments, the DNA-containing solution (1 µg DNA) was placed on a TiO_2_ thin film and irradiated with UV light at 0.5 mW/cm^2^ (UV lamp, Sankyo Denki, Kanagawa, Japan) for 10 min. Subsequently, in the visible light-responsive TiO_2_-Pt NP-mediated photocatalysis experiments, 0.2 µg of plasmid DNA were added to 100 µL of 0.5 mg/mL TiO_2_-Pt solution in 24-well plates and then placed under a visible light lamp (Classictone, incandescent lamp, 60W; Philips, Taiwan) with an intensity of 10^4^ lux (30 mW/cm^2^). UV light-responsive P25 TiO_2_ NPs (Evonik, Essen, Germany) were used for comparison. The crystal structure of the P25 TiO_2_ was a mixture of 75% anatase and 25% rutile TiO_2_; the purity was at least 99.5% TiO_2_, and the primary particle size was 21 nm, with a specific surface area of 50 ± 15 m^2^/g. Notably, the P25 TiO_2_ NPs have been used in several other antibacterial studies [2,9,12,63]. The DNA samples (i.e., photocatalyst NPs and plasmid-containing 24-well plates) of the “dark” groups in the photocatalysis experiments were covered with aluminum foil to prevent photocatalysis. Finally, competent *E. coli* cells were transformed with the photocatalyzed DNA, and the transformants were counted 18 h after plating on LB agar.

### 4.5. Blue-White Screen and Mutation Site Analysis

The blue-white screen was originally developed as a screening technique for rapid and convenient recombinant bacteria detection in vector-based molecular cloning experiments [56]. The method is based on the principle of α-complementation of the β-galactosidase gene *lac*Z; therefore, the plasmid should contain *lac*Zα (i.e., an encoding *lac*Z α-peptide, such as pBlueScript II SK^+^), whereas the *E. coli* strain (e.g., *E. coli* XL1-blue) must contain mutated *lac*Z with a deleted sequence (e.g., *lac*ZΔ*M15*). Here, the plasmid was transformed into competent host *E. coli* XL1-blue cells [30], which were then plated on LB agar plates containing 100 µg/mL ampicillin, 50 g/mL X-gal and 0.1 mM isopropyl β-d-1-thiogalactopyranoside (Sigma-Aldrich, St. Louis, MO, USA) [30]. These plates were then incubated overnight at 37 °C; after the colonies grew to an appropriate size, the plates were transferred to a 4 °C freezer. The *E. coli* cells transformed with mutant *lac*Zα-containing plasmid developed white colonies, whereas the cells transformed with functional *lac*Zα produced blue colonies. The white colonies of *E. coli* transformed with photocatalyzed plasmid DNA (pBlueScript II SK^+^) were then collected, amplified and stocked, and the plasmid DNA was further purified from these *E. coli* clones. Next, the *lac*Zα region was sequenced using the primers for the T7 promoter 5′-TAA TAC GAC TCA CTA TAG GG-3′ (reverse) and T3 promoter 5′-GCA ATT AAC CCT CAC TAA AGG-3′ (forward) located at the flanking sites of the *lac*Zα region. The primer synthesis and DNA sequencing were performed by PURIGO Biotechnology (Taipei, Taiwan) [64,65], [and the DNA sequence alignment was performed using the CLC sequence viewer 6.0.2 (CLC Bio, Qiagen, Taiwan, Taipei, Taiwan). Finally, the translation of DNA sequences into protein sequences was performed using a free online system [66].

### 4.6. Statistical Analysis

The means, standard deviations and statistics for the quantifiable data were calculated using Microsoft Office Excel 2003, SigmaPlot 10 and SPSS 19. The significance of the data was examined using one-way ANOVA, followed by post hoc Bonferroni correction. The probability of a type 1 error (α = 0.05) was identified as the threshold of statistical significance.

## 5. Conclusions

In the present study, through plasmid transformation and β-galactosidase α-complementation analyses, we determined that visible light-responsive TiO_2_-Pt NPs-mediated photocatalysis considerably reduced the number of *E. coli* transformants, suggesting that the photocatalytic reactions cause DNA damage. The DNA sequencing analyses further indicated that such DNA damage comprises three mutation types, namely nucleotide insertion, deletion and substitution. This is a pioneer study that has identified the types of mutations occurring after photocatalysis by TiO_2_-VLRPs and which may facilitate the development and appropriate usage of new-generation TiO_2_ NPs for biomedical applications.

## Supplementary Materials

The following are available online at http://www.mdpi.com/2079-4991/7/1/2/s1.

## Abbreviations

The following abbreviations are used in this manuscript:

TiO_2_	titanium dioxide
TiO_2_-Pt	platinum-containing TiO_2_
VLRP	visible light-driven photocatalyst
NPs	nanoparticles
UV	ultraviolet
ROS	reactive oxygen species
·OH	hydroxyl radicals
O_2_^−^	superoxide anions
MCS	multiple cloning site
