# Supplementary material for: Visible Light-Responsive Platinum-Containing Titania Nanoparticle-Mediated Photocatalysis Induces Nucleotide Insertion, Deletion and Substitution Mutations
Source: Nanomaterials (Basel). 2016 Dec 28;7(1):2. doi: 10.3390/nano7010002 (PMC5295192; doi:10.3390/nano7010002)
Supplement: Supplementary file 1 [file nanomaterials-07-00002-s001.pdf]

Supplementary Materials: Visible Light-Responsive Platinum-Containing Titania Nanoparticle-Mediated Photocatalysis Induces Nucleotide Insertion, Deletion, and Substitution Mutations

Der-Shan Sun, Yao-Hsuan Tseng, Wen-Shiang Wu, Ming-Show Wong and Hsin-Hou Chang

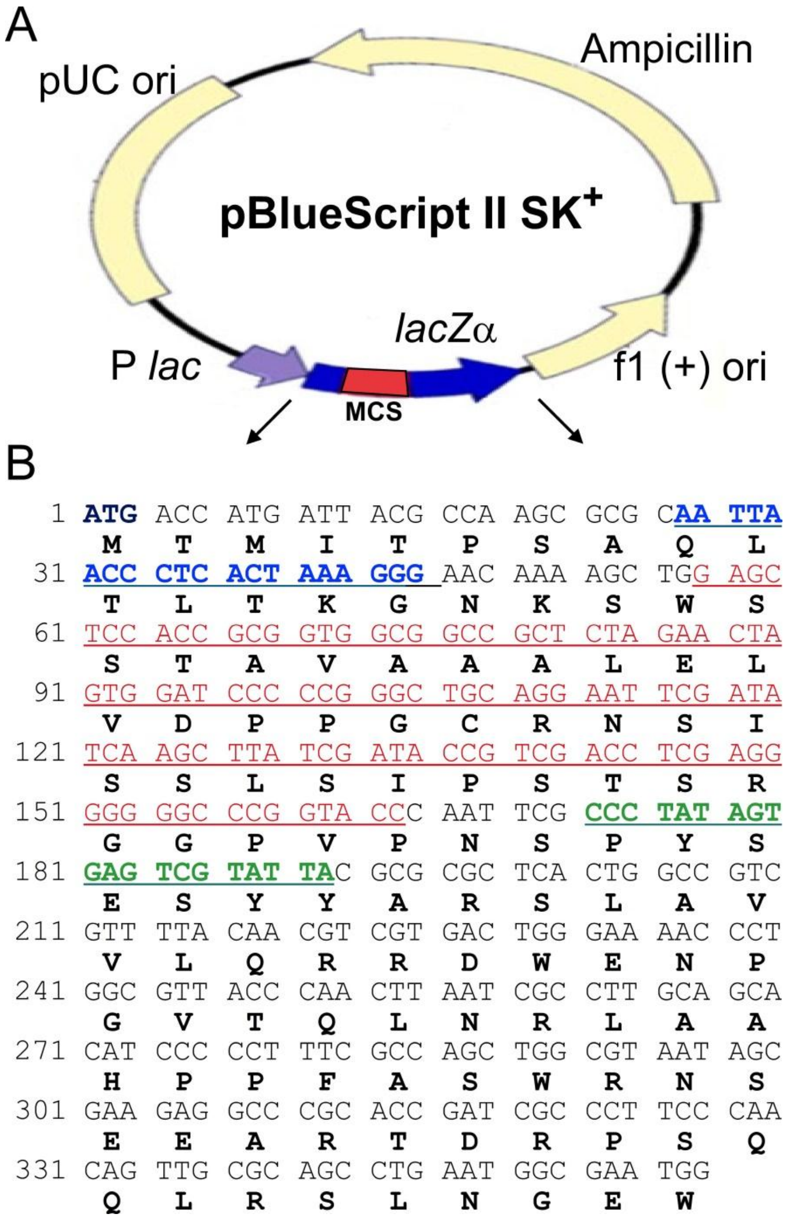

Figure S1. Plasmid map and DNA sequence of the *lac*Z α-peptide coding region. The map of the plasmid pBlueScript II SK+, a plasmid with approximately 3K base pairs, in which the coding regions of *lac* promotor (P *lac*), *lac*Z α-peptide (*lac*Zα), multiple cloning size (MCS), f1 replication origin (ori), ampicillin-resistant gene and pUC replication origin (ori) are indicated (**A**). The DNA and the corresponding amino acids sequences encoded by *lac*Zα are also indicated, in which the sequence of MCS is labeled in red, the T3 promotor primer sequence in blue and the T7 promotor primer sequence in green (**B**).

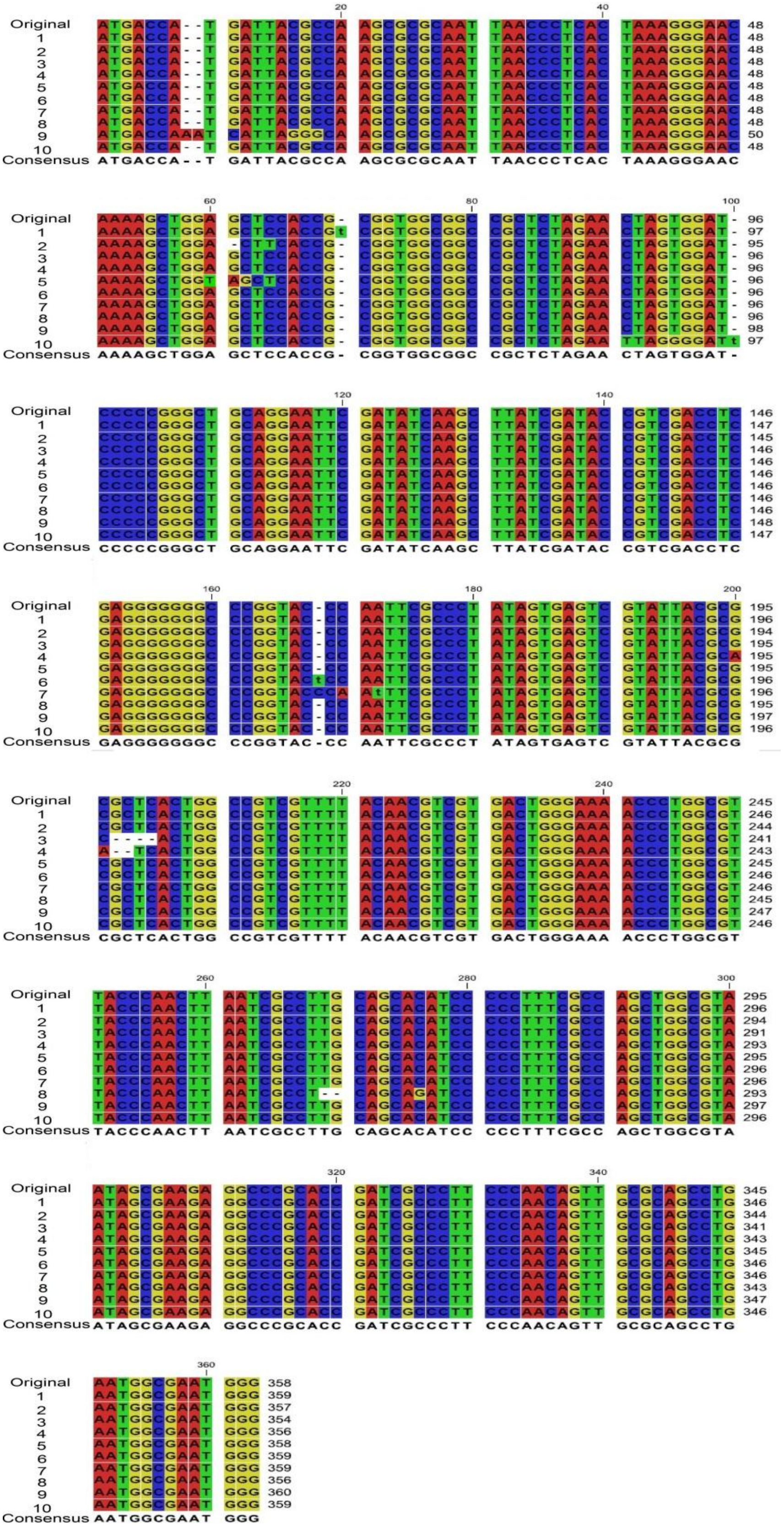

Figure S2. Mutations in the *lacZα* region. Ten mutated clones, which are shown in Figure 5, are indicated with additional highlighted color backgrounds.

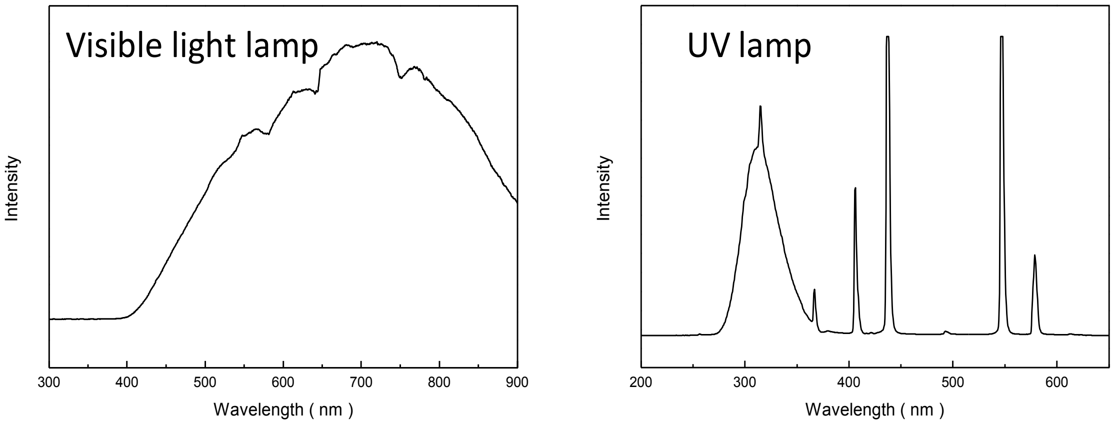

Figure S3. Spectrum analysis. Emission spectra of irradiated UV light and visible light used in this study are illustrated, in which the *x* axis indicates the relative light intensity and the *y* axis indicates the wavelength (nm).

© 2016 by the authors. Submitted for possible open access publication under the terms and conditions of the Creative Commons Attribution (CC-BY) license (http://creativecommons.org/licenses/by/4.0/).
